# Synergistic effect of nitrate exposure and heatwaves on the growth, and metabolic activity of microalgae, *Chlamydomonas reinhardtii,* and* Pseudokirchneriella subcapitata*

**DOI:** 10.1038/s41598-024-53198-7

**Published:** 2024-02-02

**Authors:** Sabiha Akter, Hamada AbdElgawad, Gerrit T. S. Beemster, Gudrun De Boeck, Jonas Schoelynck

**Affiliations:** 1 ECOSPHERE, Department of Biology, University of Antwerp, 2020 Antwerp, Belgium; 2 Integrated Molecular Plant Physiology Research Group, Department of Biology, University of Antwerp, 2020 Antwerp, Belgium; 3 Department of Botany and Microbiology, Faculty of Science, Beni-Suef University, Beni-Suef, 62521 Egypt

**Keywords:** Climate sciences, Ecology, Limnology

## Abstract

Aquatic biota are threatened by climate warming as well as other anthropogenic stressors such as eutrophication by phosphates and nitrate. However, it remains unclear how nitrate exposure can alter the resilience of microalgae to climate warming, particularly heatwaves. To get a better understanding of these processes, we investigated the effect of elevated temperature and nitrate pollution on growth, metabolites (sugar and protein), oxidative damage (lipid peroxidation), and antioxidant accumulation (polyphenols, proline) in *Chlamydomonas reinhardtii* and *Pseudokirchneriella subcapitata*. The experiment involved a 3 × 3 factorial design, where microalgae were exposed to one of three nitrate levels (5, 50, or 200 mg L^−1^ NO_3_^−l^) at 20 °C for 2 weeks. Subsequently, two heatwave scenarios were imposed: a short and moderate heatwave at 24 °C for 2 weeks, and a long and intense heatwave with an additional 2 weeks at 26 °C. A positive synergistic effect of heatwaves and nitrate on growth and metabolites was observed, but this also led to increased oxidative stress. In the short and moderate heatwave, oxidative damage was controlled by increased antioxidant levels. The high growth, metabolites, and antioxidants combined with low oxidative stress during the short and moderate heatwaves in moderate nitrate (50 mg L^−1^) led to a sustainable increased food availability to grazers. On the other hand, long and intense heatwaves in high nitrate conditions caused unsustainable growth due to increased oxidative stress and relatively low antioxidant (proline) levels, increasing the risk for massive algal die-offs.

## Introduction

Climate warming is one of the most prominent environmental stressors affecting the globe in the twenty-first century^1^. As a consequence of climate warming the intensity, frequency, and duration of heatwaves are significantly rising and pose a threat to aquatic biota^2^. Aquatic biota are also threatened by a matrix of anthropogenic stressors such as eutrophication by phosphates and nitrate pollution^1^. Moreover, rapid fluctuations in temperature due to heatwaves are increasingly likely to interact with and potentially exacerbate the effects of anthropogenic stressors^3^. It remains unclear if exposure to these stressors alters the resilience of a freshwater species to the heatwaves and how this may affect overall ecosystem functioning. Development of effective conservation solutions is critically dependent on our capacity to predict how species like microalgae in an ecosystem will respond to frequent heatwave conditions in interaction with a range of additional stressors such as nitrate pollution.

Community structures and abundance of microalgae are highly dependent on environmental parameters, including temperature and nutrient availability in the water^4^. For instance, temperature not only has an impact on photosynthesis and growth of microalgae, but also on other biological processes, such as metabolic activity, nutrient uptake, and chemical composition^5^. Furthermore, heatwaves can directly alter the efficiency of the chloroplast, and the consumption and supply of energy in photosynthetic microalgae^6^. Moreover, the morphology as well as functional activity of microalgae is affected by temperature changes^7,8^. Morphological characteristics such as cell size increase with increasing temperature^9^, which indicates an effect of temperature on the catabolic and anabolic processes of microalgae^7^. Moreover, those processes can also be affected by the duration and intensity of the heatwave as well as the availability of nutrients, including nitrate^10^_._

Anthropogenic input of nitrate/nitrogen in aquatic systems causes eutrophication and toxic algal blooms^11,12^. High nitrate levels can also change the biochemical composition of the algal cells as well as the photosynthetic activity^13^, as nitrogen is a critical component of high-value biological macromolecules, such as proteins, chlorophylls, and DNA^10,14,15^. This may affect the algal biomass production and change the lipid/protein ratio in the cell^16^ as well as the non-nitrogen compounds such as carotenoid^13^. In addition to this high nitrogen, heatwaves may have synergistic and/or additive effects on the microalgal structure and diversity^16^, and even the metabolic activity.

Algae are the energetic base of the aquatic food web^17^, therefore the fluctuations in algal growth and biomass due to high nitrogen pollution and/or heatwaves can have a substantial impact on the overall structure of ecological communities^13^. Moreover, high nitrogen pollution together with intense heatwaves may also change the metabolic activity which can affect the survival of the heterotrophs in an aquatic ecosystem^16^. An excessive abundance of microalgae in aquatic ecosystems due to high nitrate and/or heatwaves can also deplete oxygen levels at night and create hypoxic or even anoxic conditions. These hypoxic conditions may in turn affect the abundance and structure of aquatic animals such as fish communities^18^.

Co-occurrence of abiotic stressors such as changes in temperature and nutrient availability in freshwater ecosystems are common. Their occurrence and duration are likely to increase with ongoing climate change^19^. Unfortunately, the understanding of the interaction between heatwaves and nitrate in freshwater habitats is limited. In this study, we investigate the interactive effects of nitrate and a temperature surge during different heatwaves scenarios, such as short-moderate and long-intense heatwaves on algal growth, metabolic activity, and oxidative damage in *Chlamydomonas reinhardtii* and *Pseudokirchneriella subcapitata*. We hypothesize that increased nitrate levels will increase algal growth and metabolic activity^20^. The outcome of the interaction between temperature and nutrient levels therefore will affect the cell growth and this might as well change the quality of algae e.g., the concentrations of sugar, protein and antioxidants during the heatwave. However, this increased growth and metabolic activity might be limited during long and intense heatwave conditions due to limiting nutrient availability and increased oxidative stress^21^. The microalgae were exposed to different combinations of nitrate (50 mg L^−1^ NO_3_^−^) and temperatures (20, 24, and 26 °C). A 3 × 3 factorial design with 9 groups was used representing every possible scenario combination. Nitrate treatment levels were selected to reflect nutrient-deprived conditions (5 mg L^−1^ NO_3_^−^), the standard limit (50 mg L^−1^ NO_3_^−^) in European regulations for ground and surface waters, and a severely eutrophied scenario as realistic levels of nitrate in some freshwater systems can or reach beyond 200 mg L^−1^ NO_3_^–22^. A control temperature of 20 °C was chosen to represent the average summer temperature in temperate watersheds^2^, whereas 24 and 26 °C are considered as water temperatures occurring during moderate to more intense heatwaves in freshwater ponds^2^.

## Results

### Growth rate

**Figure 1:**
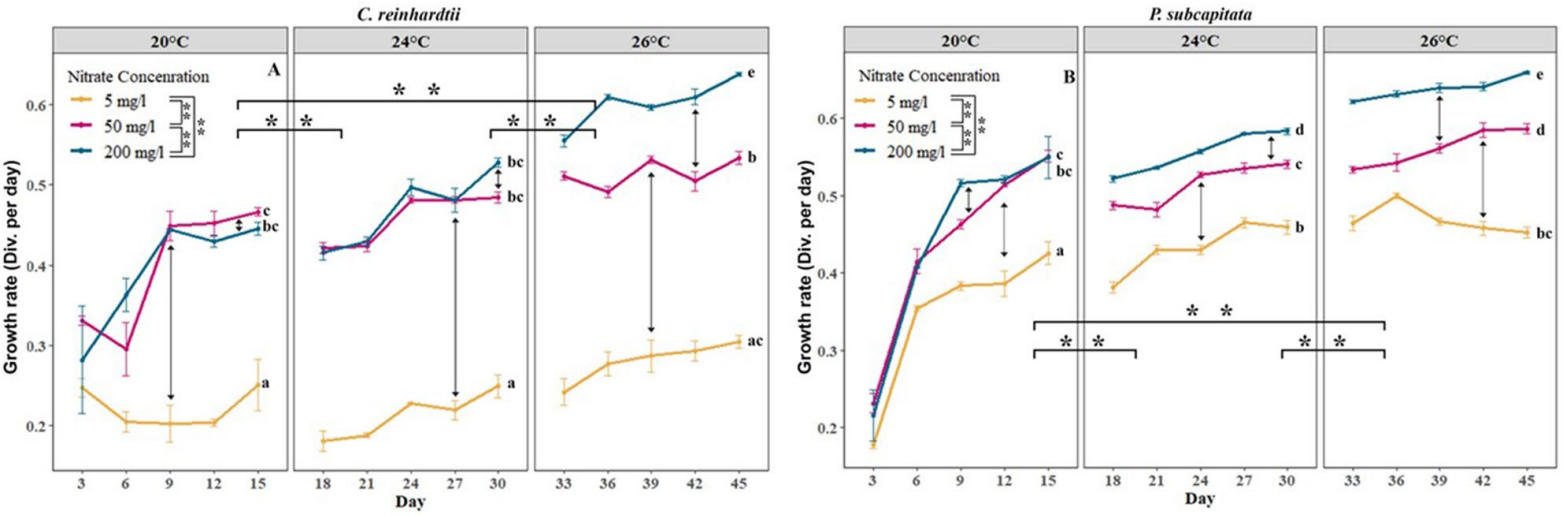
Synergic effects of nitrate and thermal acclimation on the growth rate of (**a**) *C. reinhardtii* and (**b**) *P. subcapitata*. Coloured lines represent the average growth at different nitrate levels, and error bars represent the standard error. Asterisks represent significant differences between temperatures in overall nitrate levels (with horizontal braces) and between nitrate concentrations in overall temperature (vertical braces in legend) where **represent *p* < 0.01 and *represents *p* < 0.05. Different letters represent significant differences (*p* < 0.05) in nitrate concentration of the medium within and between different temperatures.

In this study, we investigated the interactive effects of nitrate and a temperature surge on microalgal growth rate. Both short and moderate (20 to 24 °C) and long and intense (20 to 26 °C) heatwaves induced a significant increase (*p* < 0.01) in algal growth rate. Temperature accelerated the growth rate of microalgae at all nitrate concentrations and this effect was higher in microalgae cultured at the high nitrate medium (200 mg L^−1^ NO_3_^−^) than at the nitrate-deprived medium (5 mg L^−1^ NO_3_^−^) or the moderate nitrate medium (50 mg L^−1^ NO_3_^−^) (Fig. 1).

Substantial differences in the growth rate of algal cells were also observed between different nitrate media (Fig. 1). With increasing nitrate concentrations, the growth rate was significantly increased. *C. reinhardtii* and *P. subcapitata* cells cultivated in a nitrate-deprived medium proliferated slowly compared to those in a nitrate fecundated (50 and 200 mg L^−1^ NO_3_^−^) medium (Fig. 1A,B). As expected the highest growth rate was observed at the high nitrate exposure (200 mgL^−1^ NO_3_^−^) at all heatwave conditions.

Heatwaves and high nitrate levels exerted a significant positive synergistic effect (*p* < 0.01) on the growth of both algae. Heatwaves had a smaller effect on nitrate-deprived microalgae compared to moderate and high nitrate levels. Consequently, it resulted in the highest growth rate for microalgae exposed to 200 mgL^−1^ of nitrate during the long and intense heatwave treatment. Both species showed a similar positive synergistic response to heatwaves and high nitrate (Fig. 1A,B).

### Pigment content

To understand the observed growth, we analyzed the response of the photosynthetic pigments. Heatwaves significantly increased the pigment concentrations (Chl a, Chl b, carotenoid) of *P. subcapitata* and *C. reinhardtii* (Supplementary Fig. A.1). Both long and intense (20 to 26 °C) and short and moderate (20 to 24 °C) heatwaves had a significant (*p* < 0.01) positive effect on all pigment levels, except for the carotenoid in *C. reinhardtii* during the short and moderate heatwave. The temperature change from 24 to 26 °C had no additional effect on algal pigment (Supplementary Fig. A.1D).

The nitrate concentration of the medium also had a significant effect on the pigments (Chl a, Chl b, carotenoid) of the algae. Microalgae cultured in low nitrate medium had a significantly lower concentration of pigments compared to microalgae cultured at both moderate (*p* < 0.01) and high nitrate medium (*p* < 0.01) under all heatwave conditions. The difference between moderate and high N was not significant different except for Chl b at 26 °C and Chl a at 20 °C in *C. reinhardtii*.

The microalgae cultured under nitrate-deprived conditions showed a much slower augmentation in pigment content (Chl a, Chl b, and carotenoid) with temperature increase than those in nitrate-rich (50 and 200 mg L^−1^ NO_3_^−^) medium. Elevated temperatures and increasing nitrate levels only showed a synergistic impact on carotenoid concentrations in *P. subcapitata* (Supplementary Fig. A.1F). As with growth, the pigment levels of both microalgae also show a similar response but *P. subcapitata* also shows a more pronounced response than *C. reinhardtii.*

### Primary metabolic responses

As a consequence of the change in the photosynthesis process, we expected that the primary metabolite production (e.g. sugar and protein) was also altered in response to elevated temperature and nitrate concentration.

**Figure 2:**
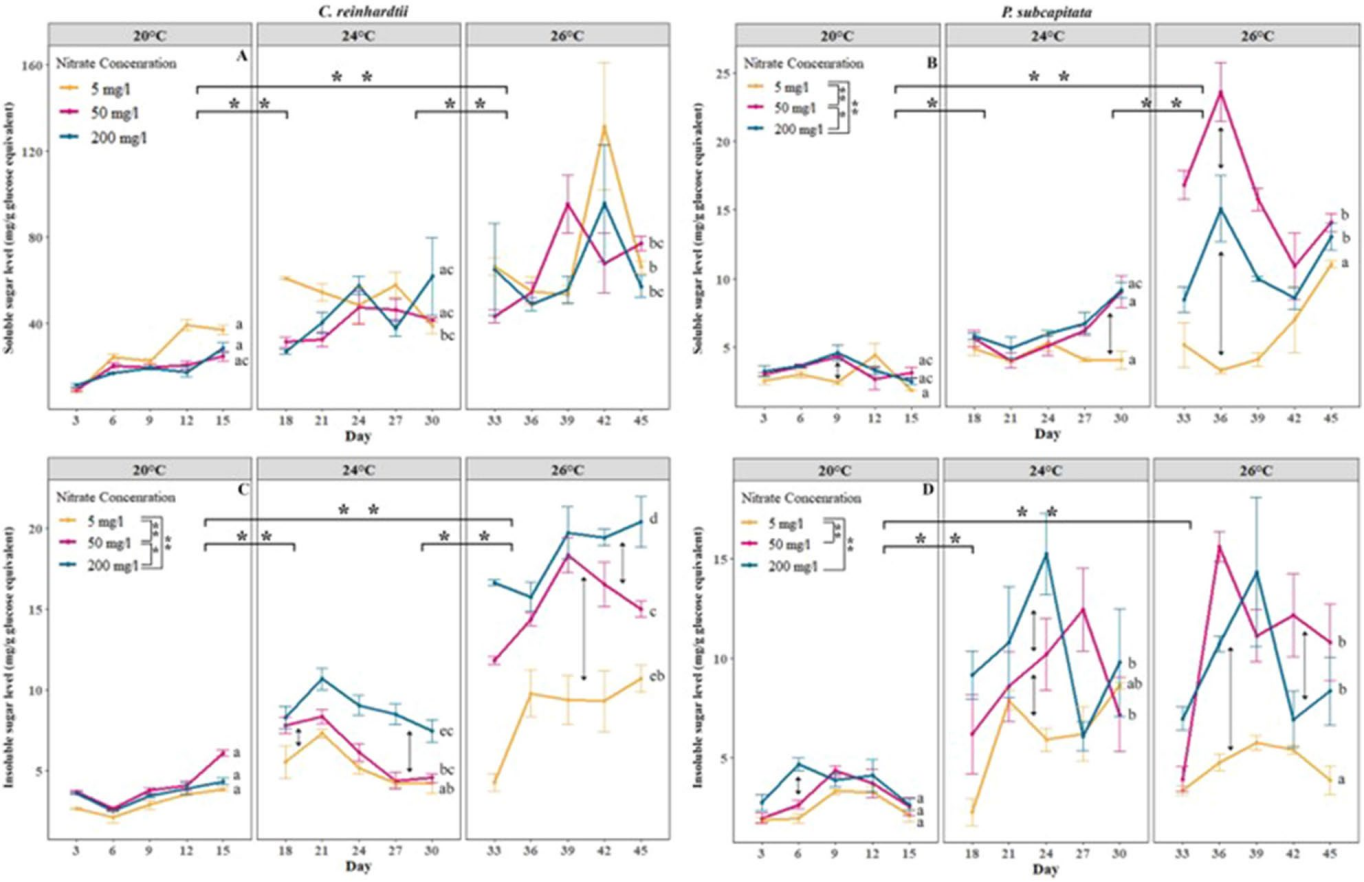
Synergic effects of nitrate and thermal acclimation treatments on sugar concentration per biomass of (**A**) Soluble sugar concentration on *C. reinhardtii* (**B**) Soluble sugar concentration on *P. subcapitata* (**C**) Insoluble sugar concentration on *C. reinhardtii* (**D**) Insoluble sugar concentration on *P. subcapitata*. Coloured lines represent the average growth at different nitrate levels, and error bars represent the standard error. Asterisks represent significant differences between temperatures in overall nitrate levels (with horizontal braces) and between nitrate concentrations in overall temperature (vertical braces in legend) where **represent *p* < 0.01 and *represents *p* < 0.05. Different letters represent significant differences (*p* < 0.05) in nitrate concentration of the medium within and between different temperatures.

*At the sugar level*, indeed*,* we found a striking change in soluble and insoluble sugar of both *C. reinhardtii* and *P. subcapitata* exposed to elevated temperatures (Fig. 2). Both long and intense (20 to 26 °C) and short and moderate heatwaves induced a gradual increase (*p* < 0.01) of sugar except for the soluble sugar concentration of *P. subcapitata* when the temperature increased from 24 to 26 °C (Fig. 2C).

Under control conditions, nitrate levels had little effect on the sugar levels. However, under heatwave conditions, the nitrate levels strongly increased sugar levels. *C. reinhardtii* and *P. subcapitata* cultured under nitrate-deprived and high nitrate exposures had a significant (*p* < 0.05) difference in soluble sugar levels per biomass only during a long and intense heatwave (Fig. 2C,D). In *P. subcapitata* the insoluble sugar levels only significantly increased after a long and intense heatwave treatment in the high and moderate nitrate medium compared to the nitrate-derived medium (Fig. 2B). Algae cultivated in a nitrate-deprived medium show a smaller increase in sugar with heatwaves, compared to those in a high nitrate medium.

*When looking at protein levels,* elevated temperature asserted a statistically significant effect on both soluble and insoluble protein concentrations in *P. subcapitata* and *C. reinhardtii*. The short and moderate heatwave reduced the soluble protein and increased the insoluble protein in both algae (Supplementary Fig. A.2). However, the temperature change from 24 to 26 °C during the extended heatwave reversed this effect.

Nitrate also shows a significant effect on the protein levels of both algae. A statistically significant increase in both soluble and insoluble protein levels occurred in *C. reinhardtii* cultured at a high nitrate medium compared to the nitrate-deprived conditions at all heatwaves, with an exception at the short and moderate heatwave (Supplementary Fig. A.1A and A.1C). However, *P. subcapitata* showed a significant increase in both protein levels in high and moderate nitrate medium compared to the nitrate-deprived condition at no heatwave (20 °C), only exceptions were observed in the soluble protein (Supplementary Fig.A.2B and A.2D).

### Oxidative stress markers

**Figure 3:**
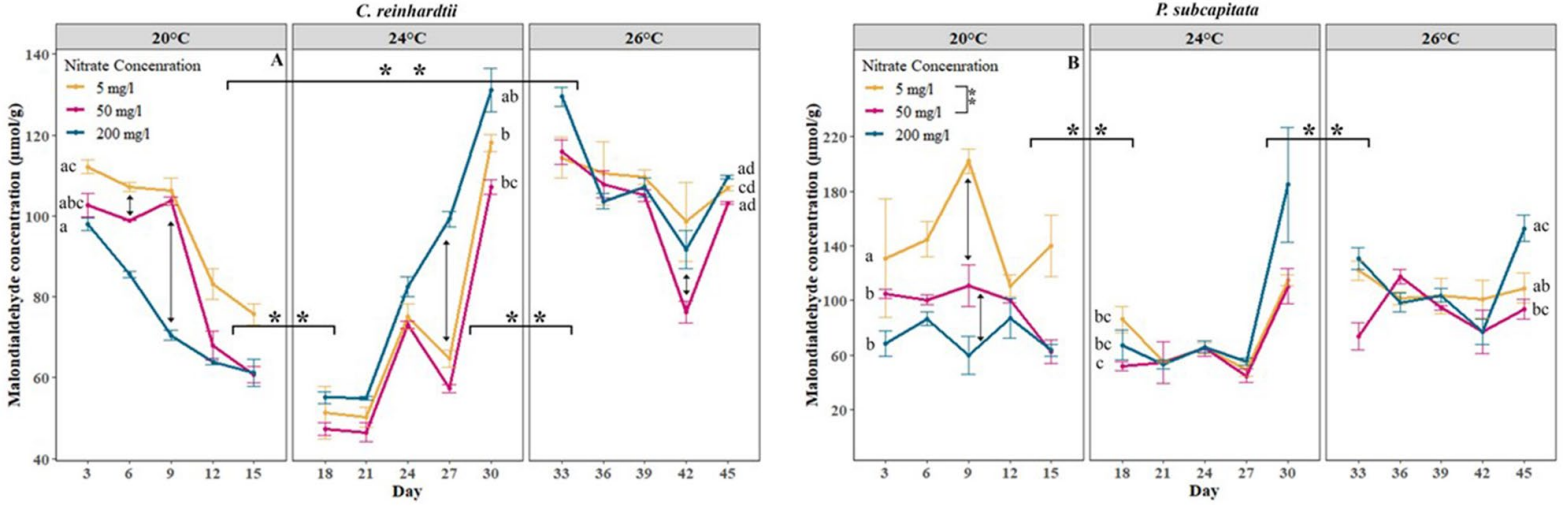
Synergic effects of nitrate and thermal acclimation treatments on malondialdehyde concentration per biomass of (**A**)* C. reinhardtii*, (**B**) *P. subcapitata*. Coloured lines represent the average Malondialdehyde concentration at different nitrate levels, and error bars represent the standard error. Asterisks represent significant differences between temperatures in overall nitrate levels (with horizontal braces) and between nitrate concentrations in overall temperature (vertical braces in legend) where **Represent *p* < 0.01 and *represents *p* < 0.05. Different letters represent significant differences (*p* < 0.05) in nitrate concentration of the medium within and between different temperatures.

Next to stimulating growth and metabolism, we expected heatwaves to cause oxidative stress. Elevated temperature significantly affected malondialdehyde (MDA) levels, a biomarker of oxidative cell damage (lipid peroxidation). A strong decrease in MDA was found at the beginning of the short and moderate heatwave (Fig. 3). But with time, the short and moderate heatwave caused a significant increase of MDA in *P. subcapitata* in all nitrate media and the MDA concentration in *C. reinhardtii* increased quite dramatically during this time (Fig. 3A,B). Long and intense heatwaves led to an increase of MDA in both microalgae.

Nitrate concentration showed a significant effect on MDA in *P. subcapitata. P. subcapitata* cultured on a nitrate-deprived medium had a significantly elevated MDA compared to high and moderate nitrate media at no heatwave (Fig. 3A). In both microalgae, the highest amount of MDA was found in the nitrate-deprived medium, and the lowest amount of MDA was found in the moderate nitrate medium (Fig. 3A,B). At low nitrate concentrations, the temperature had a bigger effect than at high nitrate concentrations. Both microalgae showed similar responses though the magnitude was different*,* with *P. subcapitata* having slightly higher MDA than *C. reinhardtii.*

### Antioxidant molecules

At the polyphenol levels, we found a significant increase (*p* < 0.01) with elevated temperature in both algae. Both heatwaves significantly increased (*p* < 0.01) algal polyphenols*,* at all nitrate levels, except in the nitrate-deprived medium during a short and moderate heatwave (20 to 24 °C) (Supplementary Fig. A.3).

Polyphenol levels were also significantly increased (*p* < 0.01) with nitrate exposure in both algal species. Microalgae cultured in a nitrate-deprived medium had lower polyphenols per unit biomass than at moderate and high concentrations, at all temperatures. In contrast, the difference between moderate or high nitrate media at any temperature condition was small and not significant. High temperature and nitrate concentration showed an additive effect on both algae (Supplementary Fig. A.3A and A.3B). The highest amount of polyphenol was found in high nitrate levels at the long and intense heatwave and the lowest amount was found in the nitrate-deprived conditions without heatwave condition.

**Figure 4:**
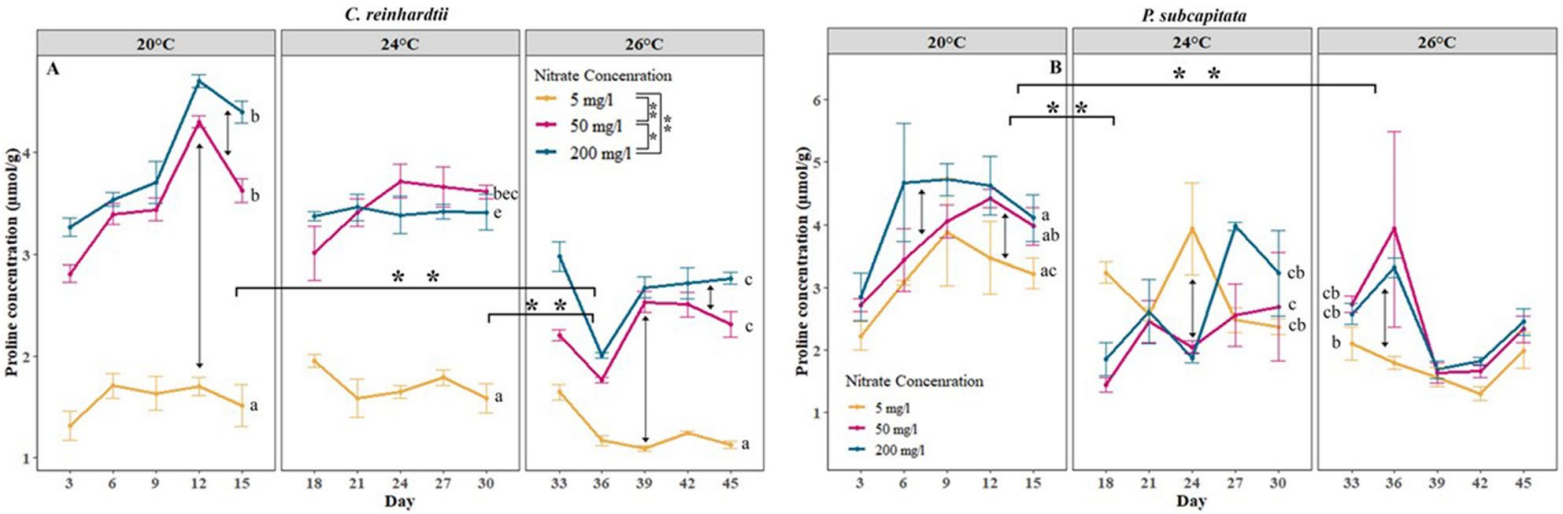
Interactive effects of nitrate and thermal acclimation treatments on proline concentration per biomass of (**A**) *C. reinhardtii,* and (**B**) *P. subcapitata*. Coloured lines represent the average proline concentration at different nitrate levels, and error bars represent the standard error. Asterisks represent significant differences between temperatures in overall nitrate levels (with horizontal braces) and between nitrate concentrations in overall temperature (vertical braces in legend) where ** represent *p* < 0.01 and *represents *p* < 0.05. Different letters represent significant differences (*p* < 0.05) in nitrate concentration of the medium within and between different temperatures.

At proline levels, we see that with elevated temperature proline levels were significantly decreased (*p* < 0.01) in both algal species**.** Long and intense heatwaves significantly decreased the proline levels in all nitrate mediums except for *C. reinhardtii* at the nitrate deprived medium and *P. subcapitata* at moderate nitrate medium (Fig. 4). The short and moderate heatwave significantly decreased the proline levels in *P. subcapitata* at moderate nitrate levels (Fig. 4B).

Proline levels were positively correlated with the nitrogen concentration of the medium (Fig. 4A,B). Proline levels in both microalgae did not show a significant difference between moderate or high nitrate medium. However, *C. reinhardtii* cultured at moderate (*p* < 0.01) or high nitrate (*p* < 0.01) media had a significantly higher proline concentration compared to microalgae cultured under nitrate-deprived conditions at all temperature conditions (Fig. 4A). On average the highest proline levels were found in the high nitrate medium at no heatwave condition and the lowest proline levels were found in the nitrate-deprived medium during a long and intense heatwave. A negative additive effect on the proline levels in both algae was observed due to the interactive action of elevated temperature and nitrate concentration.

## Discussion

Addressing multi-stressor effects is a necessary way forward to approach ecological reality. Unfortunately, even knowledge of the interactions between prevalent stressors like nitrate and heatwaves remains limited. In this study, we examined the combined effects of nitrate exposure and heatwaves on growth, metabolic responses, oxidative stress, and antioxidant activity of *P. subcapitata* and *C. reinhardtii*. Growth, primary metabolites (sugar, protein), and antioxidants (polyphenols, proline) of microalgae significantly increased with elevated nitrate availability during the short and moderate heatwave. This high growth and metabolite levels were limited at high temperatures in nitrate-deprived conditions but synergistically (growth) and additively (metabolites) increased in high nutrients under long and intense heatwave conditions. Heatwaves induced oxidative stress whereas antioxidant level were up at short and moderate heatwaves but they were reduced at long and intense heatwave.

Nitrate and heatwaves interacted synergistically and/or additively during our simulation to increase the growth and metabolic levels of both microalgae. Duration and intensity of the heatwave was the most influential variable affecting the chemical composition and growth of microalgae. These findings are in line with previous research, e.g., Yoshioka et al., (2020)^24^ also found temperature as the most influential variable for microalgae. Due to the temperature dependency of enzymatic reactions and catalytic processes^13,23^, primary photochemical reactions increase with heatwaves which causes high growth and metabolic activity at high temperatures in heatwave conditions. Fast-growing cells at a high temperature also have high requirements for limiting nutrients like N and P^24^. Growth was significantly lower in the nitrate-deprived medium even after long and intense heatwaves, and a synergistic increase in growth was observed at high nitrate during long and intense heatwaves. Moreover, the low nitrogen in the nitrate-deprived medium limited the chlorophyll concentrations which is also responsible for the low growth rate^25^. At the same time, the highest carotenoid concentration in high nitrate levels during the heatwaves may work as a protection against photodamaging and help to maintain the high growth rate^26^. This was necessary as eutrophication not only accelerated the synergistic effect of long and intense heatwaves and nitrate on growth and metabolites but also induced oxidative stress on the algae.

Excessive growth of microalgae due to high nitrate concentrations is considered a primary symptom of eutrophication. It may have severe impacts such as a loss of submerged aquatic vegetation, oxygen depletion, and imbalanced food webs^27^. On top of the eutrophic conditions with rich-N, high temperature during heatwaves increases the magnitude of algal growth in these conditions. Water directives and legislation try to control eutrophication by limiting N, but their work might be counteracted by the effect of more frequent heatwaves.

Moreover, photosynthetic use of inorganic carbon by excessive microalgae due to high nitrate and temperature may create alkaline conditions in the upper layer of the water column^27^. High alkaline conditions of those eutrophic ecosystems can damage the chemosensory ability of organisms. A high abundance of microalgae during long and intense heatwaves may also create shading for the lower water column, reduce the depth of the critical layer, and produce excessive dissolved organic carbon (DOC) in the lower water column^28,29^. This high DOC in the lower water column can then reduce the pH which may neutralize alkaline conditions in the upper water column during the long and intense heatwaves but this hypothesis mainly depends on the depth and water circulation between different water compartments of an ecosystem. If water circulation between different water layers is not sufficient then the organic material in the lower critical layer will additionally produce a hypoxic condition with high DOC and humid substances (HS). But in both scenarios, high HS can attenuate photosynthetically active radiation, which may limit benthic primary production and growth of submerged vegetation despite a high amount of nutrients. Moreover, increasing pH and reducing inorganic carbon availability in the upper layer of the water column along with the hypoxic condition of the lower layer of the water column can also affect the algal community structure. The latter condition favors species like cyanobacteria which can use bicarbonate instead of carbon as an energy source^29^.

Furthermore, the physiological disturbance of microalgae in nitrate-deprived and high nitrate levels was confirmed with high lipid peroxidation markers which is the primary indicator of oxidative stress^30^. During the short and moderate heatwaves, microalgae may have coped with the physiological disruption using high levels of polyphenols and proline as a natural antioxidative response against oxidative stress. High MDA and low proline concentrations in the long and intense heatwave treatment may indicate the breakdown of natural defenses against oxidative stress. Indeed Yuan-Chun and Tse-Min (1999)^31^ also showed that the long-term effects of temperature are to reduce the accumulation of proline. They found a significant decrease in the accumulation of these non‐enzymatic ROS scavengers in *G. tenuistipitata* after exposure from 20 to 35 °C. Those antioxidant levels along with high growth and energy at high nitrate during the heatwaves may also alter the availability of food and energy to the primary consumer to cope with this stress condition.

The synergistic effects of heatwaves and nitrate exposure on the primary and secondary metabolism of the microalgae may alter the overall energy flow in the aquatic ecosystem. Sugar in microalgae is one of the main primary energy sources for aquatic animals. With nitrate concentration, heatwaves synergistically increased both the soluble and insoluble sugar in microalgae. Similar to our study, Juneja et al., (2013)^25^ also found an increase in sucrose (57%) in microalgae when exposed to 38 °C. The high sugar levels in microalgae during the heatwaves may help herbivores to fulfill the higher energy demand at higher temperatures^32^. Like sugar, algal protein is an indicator of the primary metabolism of algae. Algal protein is the main source of nitrogen and essential amino acids for aquatic animals. Nitrogen is an indispensable part of each polypeptide in protein structures^21,33^. A dramatic increase in protein levels with high nitrate concentration was expected, yet we observed a minimal change in both soluble and insoluble protein levels with different nitrate in the medium, similar to the previous observation made by Tarazona et al., (2021)^25^. Araújo and Garcia (2005)^34^ found the protein content to be unaffected at lower temperatures (20 °C and 25 °C) compared to high temperatures (30 °C) in the diatom *Chaetoceros cf*. In our study, nitrate and temperature together had a significant effect, demonstrating an additive effect on algal protein concentration. The high amount of protein and sugar as energy in the food can affect the physiological activity of primary consumers during short and moderate heatwave conditions as those are two essential nutrients for growth and metabolism^35^. High nitrate and temperature can increase the availability and also the quality of food for grazers as these parameters increase the sugar and protein content of the microalgae which may help the grazer to cope with the increased energy demand due to the short and moderate heatwave in the eutrophic ecosystem.

Along with the high energy content of algae, high antioxidant activity will also help the primary consumer cope with extreme heatwave conditions. Ahmadifar et al. 2021 and Madeira et al. 2013^36,37^ reported that dietary polyphenols can improve the antioxidant defenses, immune response, disease resistance, reproductive performance, and growth performance of aquatic fish. On the other hand, Ahmadifar and co-workers^38^ showed that at high temperatures proline can increase the immune response against bacterial infection. High amounts of proline contribute to oxidative stress tolerance by scavenging ROS and other free radicals^39^. Based on our findings, we conclude that a synergistic increase of non‐enzymatic ROS scavengers like proline during the short and moderate heatwave in a eutrophic ecosystem, will improve the antioxidants defense and the survival of the grazers as the oxidative stress of invertebrates and fish increases with the temperature^36^. During long and intense heatwaves, oxidative stress (lipid peroxidation) increased, and antioxidants like proline decreased. In long and intense heatwaves with high nitrate conditions, primary producers like microalgae may become unable to provide enough antioxidants to the primary consumers higher in the food web.

## Conclusion

Our findings demonstrated that nitrate and temperature interacted synergistically on growth rate and the biochemical composition of microalgae. Heatwaves and high nitrate cause positive synergistic effects on algal growth which may promote hypoxic conditions in an aquatic ecosystem. This positive synergistic interaction during the short and moderate heatwaves also increased the quality of the microalgae as food with high sugar, protein, and antioxidant capacity, which may influence the overall primary consumer response in the ecosystem towards eutrophication. However, severely elevated nitrate levels lead to unsustainable and poor-quality microalgae with high oxidative stress and relatively low antioxidants during long and intense heatwaves. Further research on the performance of primary consumer response towards food quality during eutrophication and different heatwave conditions is needed.

## Materials and methods

### Experimental species and maintenance

Geographically and ecologically relevant algal species*, C. reinhardtii,* and *P. subcapitata* were selected for this study. *C. reinhardtii* is a single-cell eukaryote microalga found in freshwater throughout the world. It has been extensively studied because of its adaptability to cope with different environments and its fast growth with a 5-h generation time^40,41^. It can change from haploid to diploid during states of nitrogen deprivation and it can also use alternative carbon sources in the absence of light. *P*. *subcapitata* formerly known as *Raphidocelis subcapitata* is a dominant species of green microalgae in freshwater ecosystems. *P. subcapitata* is widely used for bioassays in toxicological risk assessments^42^ and is commonly used as a bioindicator species to assess the levels of nutrients or toxic substances in freshwater environments.

### Experimental setup

**Figure 5:**
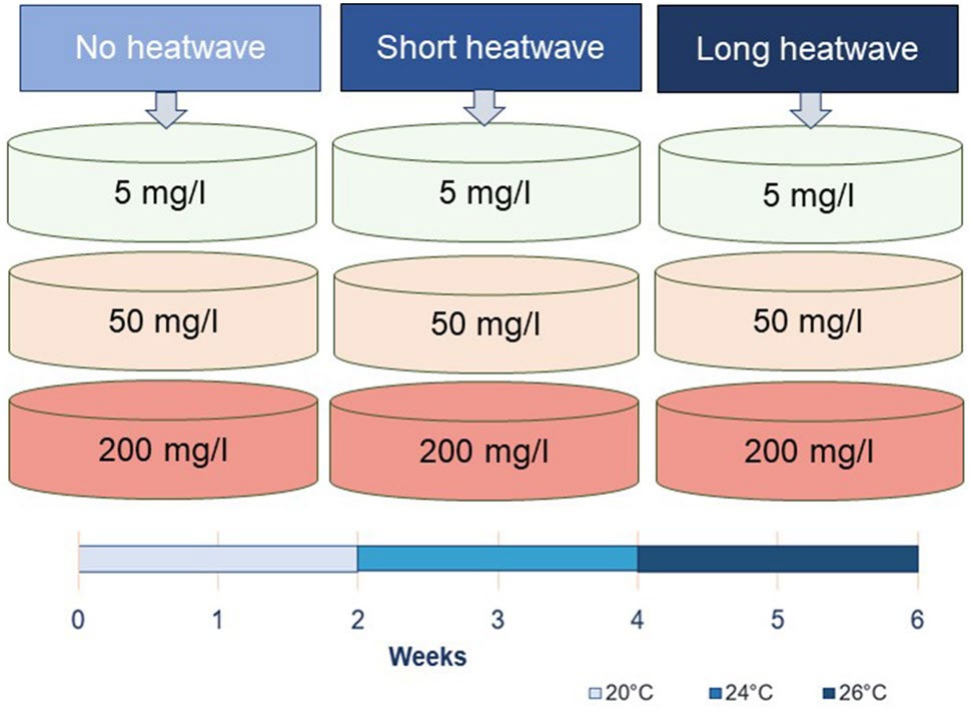
Schematic display of the experimental design including all 9 possible scenarios and timeline of the different heatwave scenarios.

Axenic strains of the microalgae *C. reinhardtii* (SAG 11-32a) and *P. subcapitata* (CCAP278/4) were obtained from standard cell cultures at the ECOSPHERE lab, University of Antwerp, Belgium. Those microalgae were cultivated in 1000 mL glass flasks under continuous cool-white, fluorescent lamps (≈100 μmol photons/m^2^ s^−1^) on a 14:10 h light–dark cycle. Continuous airflow through a filter was ensured to keep above 80% of dissolved oxygen (DO) and also equal distribution of cells within the culture. Modified Bold’s basal medium (BBM) medium with CaCl_2_ × 2H_2_O, MgSO_4_.7H_2_O, K_2_HPO_4,_ KH_2_PO_4,_ NaCl, Na_2_EDTA, ZnSO_4_ × 7H_2_O, H_3_BO_3,_ MnCl_2_ × 4H_2_O, CoCl_2_ × 6H_2_O, CuSO_4_ × 5H_2_O, (NH_4_)_6_Mo_7_O_24_ × 4H_2_O, FeSO_4_ × 7H_2_O was used for optimal growth. Before the onset of the experiment *C. reinhardtii* and *P. subcapitata* were acclimated to 20 °C over 7 days in a modified BBM medium supplemented with 50 mg NO_3_^−^ per liter (50 mg L^−1^ NO_3_^−^) at the same light/dark cycle as the experiment. After this adjustment period, the combined effect of water temperature and nitrate on the growth, metabolic activity, oxidative stress, and antioxidant activity of microalgae was investigated. The experiment was conducted in the controlled environment of a climate chamber for 6 weeks where the microalgae were assigned to one of three nitrate levels (low: 5 mg L^−1^ NO_3_^−^, moderate: 50 mg L^−1^ NO_3_^−^, high: 200 mg L^−1^ NO_3_^−^; actual measured values of 5.7 ± 1.0 mg L^−1^ NO_3_, 50 ± 3.0 mg L^−1^ NO_3_^−^, and 200 ± 8.0 mg L^−1^ NO_3_^−^, respectively, average ± SD, N = 17) at 20 °C for two weeks (Fig. 5). After that period, a short (2 weeks) and moderate heatwave of 24 ± 0.5 °C was imposed on all nitrate treatments. The heatwave period was prolonged for another 2 weeks and intensified up to 26 ± 0.5 °C, creating a long and intense heatwave (Fig. 5). Each combination of treatments was run in three randomly selected replicates.

Throughout the experiment, the pH of the medium was checked frequently with a pH meter (744 Metrohm) to maintain levels at 8.5 ± 0.5. The nitrate concentration of the medium was checked using a VISOCOLOR ECO nitrate colorimetric test kit (Macherey–Nagel, Germany) in a compact photometer PF-12 plus (Macherey–Nagel) each time before microalgae were added to the medium to ensure the appropriate nitrate concentration for the treatment. Initially, 5 × 10^5^ cells/mL were incubated in a 400 mL medium in a 1000 mL glass bottle for each treatment. Every 3^rd^ day ~ 2–4 × 10^6^ cells of microalgae were harvested, the remaining medium was removed by centrifugation with a high-performance centrifuge (Avanti™ J-25, USA), and cells were placed into fresh medium to maintain the desired nitrate concentration. The remaining cells in the old medium were collected to measure the growth rate, and samples were collected, centrifuged, and stored at -80 °C for the determination of biochemical composition, oxidative stress, and antioxidant activity.

### Growth and biochemical responses of algae

#### Growth rate

To calculate the growth rate, the initial number of cells and the number of cells after three days were determined using a Coulter counter (Multisizer™ 3). Before the growth rate calculation growth within every 3 day-period was measured and that proved to be quite linear in each exposure condition with R^2^ between 0.9819 and 0.9941 for *P. subcapitata* and R^2^ between 0.8872 and 0.9247 for *C. reinhardtii* (Supplementary Fig. A.4). The growth rate (µ) as growth rate division (Div.) per day was calculated using the following formula.

$$\mu \, = {\text{log}}_{{2}} \left( {{\text{C}}_{{1}} /{\text{C}}_{0} } \right)/\Delta {\text{t}}$$

where C_0_ = Initial number of the cell of microalgae mL^−1^, C_1_ = Number of the cell of microalgae mL^−1^ and Δt = Time between two calculations (days).

#### Sample preparation

To quantify pigments, metabolic response, oxidative stress, and antioxidative mechanisms, microalgae stored at − 80 °C were weighed and homogenized in 80% ethanol ^43^. The samples were centrifuged for 15 min at 3000×*g* to collect the supernatant and pellet. Small supernatant subsamples were immediately used for pigment determination and the rest of the supernatant was stored at − 80 °C for future determination of malondialdehyde, polyphenols, proline, soluble sugar, and soluble protein. The pellets were kept at − 20 °C for insoluble sugar and insoluble protein determination.

#### Pigments

Pigments in supernatants from the homogenized samples were spectrophotometrically measured at wavelengths 663, 649, and 470 nm using a microplate reader (Synergy Mx, Biotek Instruments Inc., Vermont, VT, USA). The pigment content was calculated according to Wellburn (1994) ^43^.

#### Metabolites

##### Soluble and insoluble sugar

Supernatants from the homogenized samples were used to determine the soluble sugar content while the pellets were used for insoluble sugar. The supernatant contains the soluble sugars glucose, fructose, and sucrose as well as other compounds, such as chlorophyll and lipids. To remove the latter compounds, chloroform was used while hydrochloric acid was used to extract the insoluble sugar from the pellet. According to Hansen and Moller, 1975^44^. The Anthrone assay was used to determine both soluble and insoluble sugar content using glucose as standard.

##### Soluble and insoluble proteins

Both soluble and insoluble proteins were measured according to the Folin-Lowry method. To extract soluble protein from the supernatant, 10% trichloroacetic acid (TCA) was added and the precipitate was redissolved in 1 M sodium hydroxide (NaOH), while for insoluble protein, the pellet was washed with ethanol, ethanol/chloroform (3:1 v/v), ethanol/diethyl ether (3:1 v/v) and ether, to remove the phenolic compound and also redissolved in 1 M NaOH. Absorbance was read at 650 nm against the standard curve of bovine serum albumin (BSA) powder using a microplate reader.

#### Oxidative stress markers

Malondialdehyde (MDA) is one of the most prevalent by-products of lipid peroxidation during oxidative stress. Malondialdehyde was measured as an indicator of oxidative stress using Thiobarbituric acid (TBA). TBA was added to the supernatant to produce the pinkish-red chromogen, thiobarbituric acid-malondialdehyde (TBA-MDA). The absorbance was measured at wavelengths 440, 532, and 600 nm using a microplate reader.

#### Osmoprotectant and antioxidants

##### Proline

Free proline was determined in the supernatant using ninhydrin (2,2-dihydroxyindane-1,3-dione) and anhydrous acetic acid, according to Bates, (1973) ^45^. Absorbance was read at 520 nm against a standard curve of L-proline using a microplate reader assay.

##### Polyphenols

The Folin-Ciocalteu assay was used to determine the total phenol content in the supernatant^46^. The absorption was read at 725 nm against a standard curve of gallic acid by using a microplate reader.

### Statistical analyses

Data analyses were carried out using R Studio (version 3.6.0). ANOVA with linear mixed effects (lme) models was performed to discern the effect of acclimation temperature (three-level, fixed factor) and nitrate concentration (three-level, fixed factor) and the interaction between two stresses. To check the homogeneity and normal distribution of data, Levene’s and Shapiro's tests were run. The ANOVA assumptions were checked, and Tukey’s post hoc tests were run to determine statistical differences among treatment groups and fixed factor levels. Statistical significance was accepted at a probability level less than or equal to 0.05 (*p* < 0.05). Data were presented as average value ± standard error (SE).

### Supplementary Information

Supplementary Information.

## Data Availability

The authors highly appreciate and state that data will be available from the corresponding author on request.
