# Supplementary material for: Synergistic effect of nitrate exposure and heatwaves on the growth, and metabolic activity of microalgae, Chlamydomonas reinhardtii, and Pseudokirchneriella subcapitata
Source: Sci Rep. 2024 Feb 2;14:2764. doi: 10.1038/s41598-024-53198-7 (PMC10837129; doi:10.1038/s41598-024-53198-7)
Supplement: Supplementary file 1 — Supplementary Information. [file 41598_2024_53198_MOESM1_ESM.pdf]

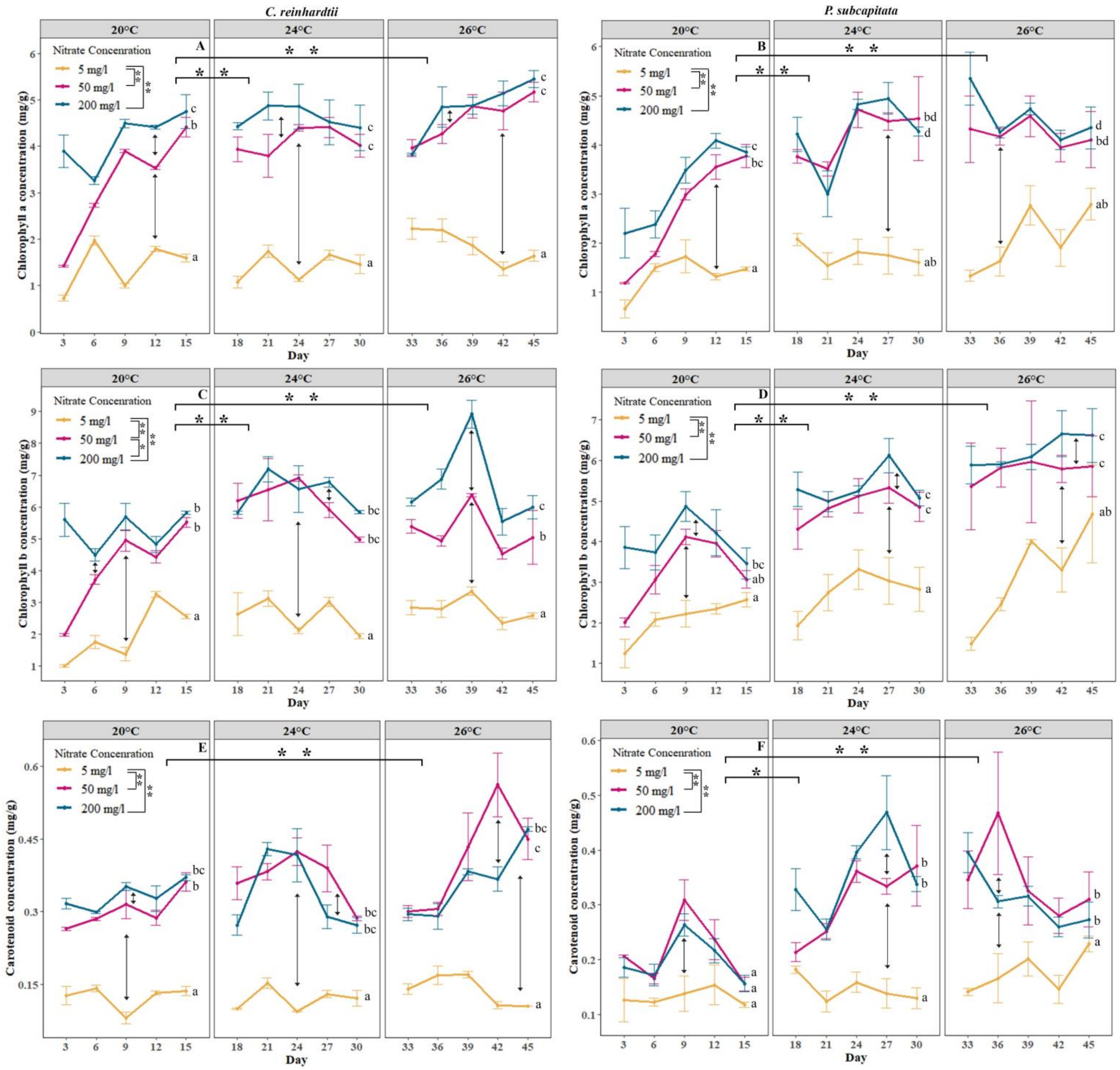

Figure A.1: Synergic effects of nitrate and thermal acclimation treatments on pigment concentration per biomass of (A) Chlorophyll a concentration in *C. reinhardtii* (B) Chlorophyll a concentration in *P. subcapitata*, (C) Chlorophyll b concentration in *C. reinhardtii* (D) Chlorophyll b concentration in *P. subcapitata* (E) Carotenoid concentration in *C. reinhardtii* (F) Carotenoid concentration in *P. subcapitata***.** Coloured lines represent the average growth at different nitrate levels, and error bars represent the standard error. Asterisks represent significant differences between temperatures in overall nitrate levels (with horizontal braces) and between nitrate concentrations in overall temperature (vertical braces in legend) where ** represent $p<0.01$ and * represents $p<0.05$. Different letters represent significant differences ($p<0.05$) in nitrate concentration of the medium within and between different temperatures.

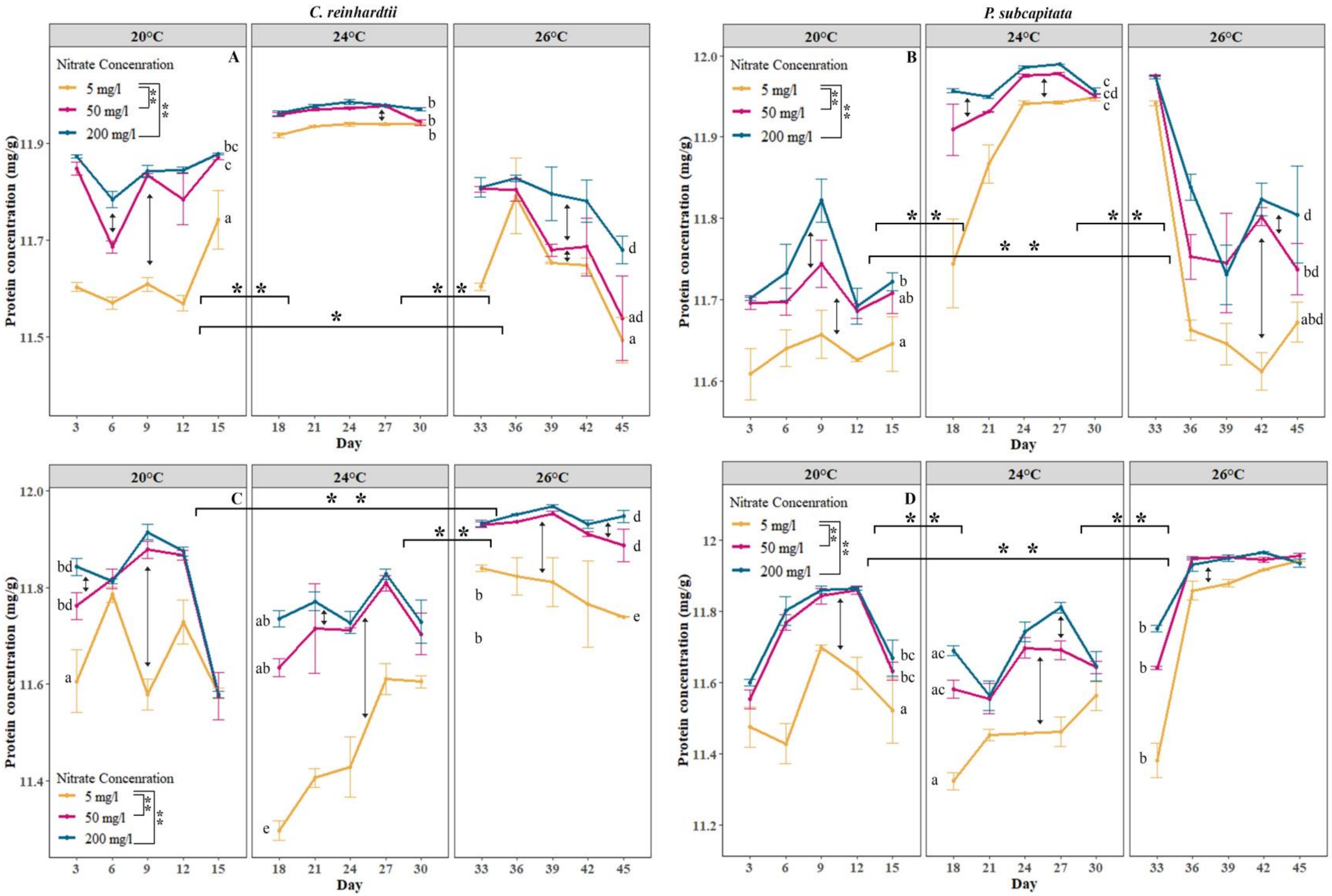

Figure A.2: Synergic effects of nitrate and thermal acclimation treatments on protein concentration per biomass of algae of (A) Insoluble protein concentration on *C. reinhardtii* (B) Insoluble protein concentration on *P. subcapitata*, (C) Soluble protein concentration on *C. reinhardtii*, (D) Soluble protein concentration on *P. subcapitata*. Coloured lines represent the average growth at different nitrate levels, and error bars represent the standard error. Asterisks represent significant differences between temperatures in overall nitrate levels (with horizontal braces) and between nitrate concentrations in overall temperature (vertical braces in legend) where ** represent p<0.01 and * represents p<0.05. Different letters represent significant differences (p<0.05) in nitrate concentration of the medium within and between different temperatures.

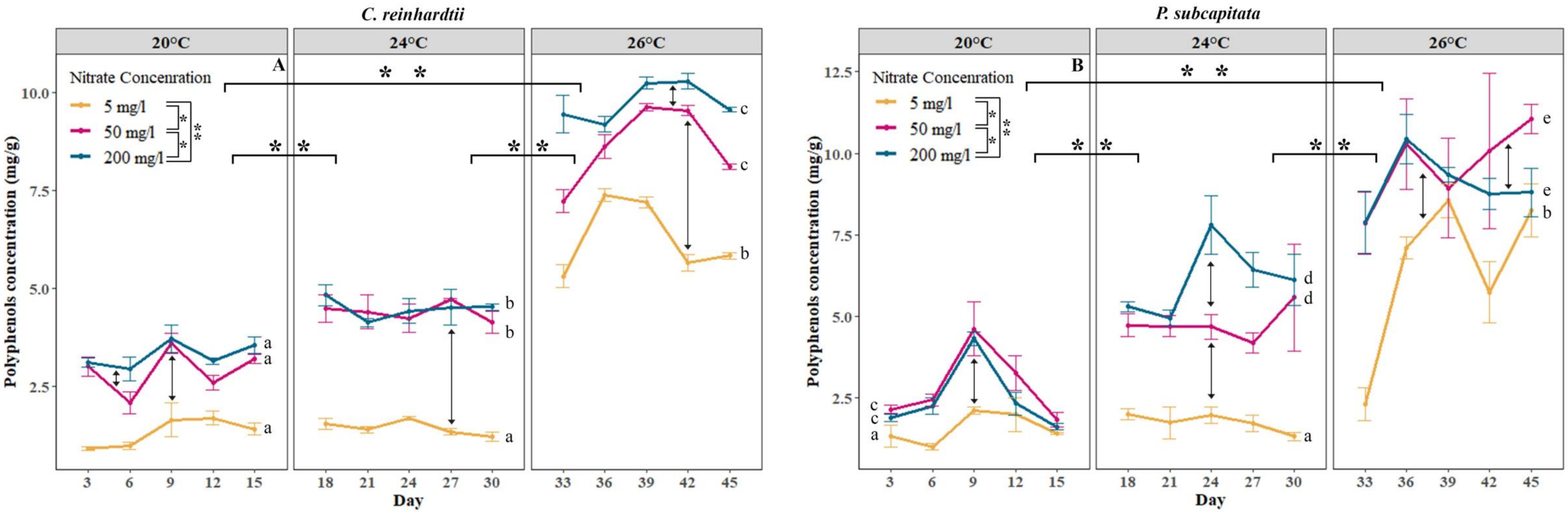

Figure A.3: Interactive effects of nitrate and thermal acclimation treatments on polyphenols concentration per biomass of (A) *C. reinhardtii*, and (B) *P. subcapitata*. Coloured lines represent the average polyphenols concentration at different nitrate levels, and error bars represent the standard error. Asterisks represent significant differences between temperatures in overall nitrate levels (with horizontal braces) and between nitrate concentrations in overall temperature (vertical braces in legend) where ** represent $p<0.01$ and * represents $p<0.05$. Different letters represent significant differences ($p<0.05$) in nitrate concentration of the medium within and between different temperatures.
